# Etymologia: Eptesicus fuscus

**DOI:** 10.3201/eid1112.ET1112

**Published:** 2005-12

**Keywords:** etymologia, bat, *Eptesicus fuscus*

## [ep´tes-ə-kəs fəs-kəs]

The big brown bat (Figure). From the Greek *epten*, "I fly," plus *oikos*, "house," and the Latin *fuscus*, "dusk." A nocturnal, insectivorous bat, *Eptesicus fuscus* females separate after mating into maternity colonies that are frequently found in attics of buildings or other manmade locations, since they prefer warmer temperatures in which to raise their young.

**Figure:**
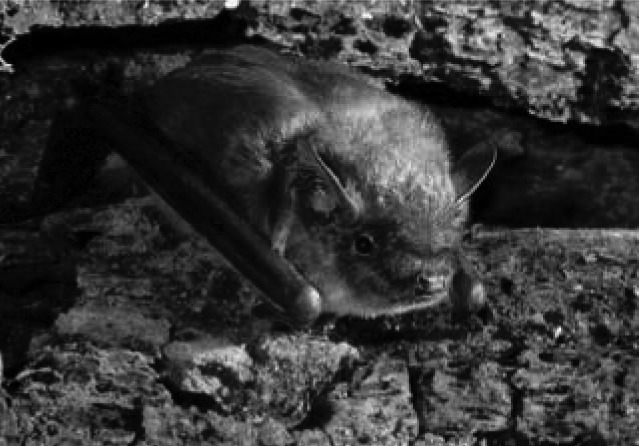
Photograph courtesy of Ivan Kuzmin.

**Sources:** McElhinny T. A mammalian lexicon. [cited 2005 Oct 13]. Available from http://www.msu.edu/~mcelhinn/zoology/mammalwords.htm; Webster's Third New International Dictionary (unabridged). Springfield (MA), 1993; and wikipedia.org.

